# Examining the relationship between health literacy and individual and sociodemographic factors in secondary school students

**DOI:** 10.1007/s10389-023-01836-1

**Published:** 2023-02-02

**Authors:** Dominik Pendl, Katharina Maria Maitz, Barbara Gasteiger-Klicpera

**Affiliations:** 1 Institute of Education Research and Teacher Education, Inclusive Education Unit, University of Graz, Merangasse 70/2, 8010 Graz, Austria; 2 Research Center on Inclusive Education, University of Graz, Strassoldogasse 10/2, 8010 Graz, Austria

**Keywords:** Subjective health literacy, eHealth literacy, Secondary school students, Individual factors, Sociodemographic factors

## Abstract

**Aim:**

Health literacy (HL) is an important factor in health promotion, especially regarding children and adolescents. The present study aims to identify the individual and sociodemographic factors related to secondary school students’ HL. This should make it possible to find specific strategies to improve HL.

**Subject and methods:**

Data on the sociodemographic background (migrant background, number of books at home and spoken language at home), self-efficacy, online reading behaviour, subjective HL (adapted version of the European Health Literacy [HLS-EU] questionnaire) and the eHealth Literacy Scale (eHEALS) of 544 Austrian secondary school students (age 11–16, 46% girls) were collected. Regression analyses were conducted to test the hypotheses regarding those factors which influence students’ subjective HL and eHL.

**Results:**

Students subjective HL (*M* = 3.79, *SD* = .63) and eHL (*M* = 3.46, *SD* = .77) scores were rather high on average. Subjective HL was predicted by age, gender and online reading behaviour. However, only students’ online reading behaviour was highly significant and was found to be the most influential predictor of subjective HL. Age and online reading behaviour were also found to be highly significant predictors regarding eHL.

**Conclusion:**

Students’ online reading behaviour and age are important factors linked to HL. Educational activities may serve to reduce health inequalities by fostering relevant internet skills, i.e. the skills needed to facilitate effective and critical use of internet information.

## Background

In Europe, health literacy (HL) has continued to gain significance since the 1990s (Ganahl and Pelikan 2016) and is now seen as an important factor in widespread health promotion and in the provision of equal opportunities in the healthcare system (Nutbeam 2000). The initial definitions of the concept derive from the USA (Ad Hoc Committee on Health Literacy for the American Council on Scientific Affairs 1999) and focus on the development of basic functional skills, such as the basic reading and numeracy skills needed to understand health information (Mårtensson and Hensing 2012; Parker et al. 1995) and to “function” in the healthcare system (Ad Hoc Committee on Health Literacy for the American Council on Scientific Affairs 1999). This initial focus on the functional aspects of HL was subsequently developed and augmented (Berkman et al. 2010), and in addition to functional literacy, the concept came to incorporate communicative/interactive and critical literacy (one of the best known concepts in this regard is that of Nutbeam [2000]). Communicative/interactive literacy refers to the cognitive and social skills needed in *applying* information, while critical literacy refers to the cognitive and social skills employed in *questioning* information. In addition, the move from a purely individual perspective of HL towards a more social perspective, one which was capable of taking public health aspects into account (Freedman et al. 2009; Nutbeam 2008), then led Sørensen et al. (2012) to the following definition of HL: HL is “linked to subjective literacy and entails people’s knowledge, motivation and competences to access, understand, appraise, and apply health information in order to make judgments and take decisions in everyday life concerning healthcare, disease prevention and health promotion to maintain or improve quality of life during the life course” (Sørensen et al. 2012, p. 3). Based on this definition, throughout this paper we use the term “subjective” HL. This latter term may be seen as a multidimensional construct, and is one which is now frequently used in public health research (Okan et al. 2017; Sørensen et al. 2012). It distinguishes itself from the former medical understanding of HL, in that it no longer focuses on functional skills alone (Sørensen 2020). The ongoing COVID-19 pandemic has made HL more important than ever (Sentell et al. 2020), as it is closely linked to health-related behaviour (Ormshaw et al. 2013). It has been found that people with insufficient HL exhibit a higher level of alcohol consumption (HLS-EU Consortium 2012), have relatively little knowledge of medicines, lack the ability to deal with pharmaceuticals adequately (Berkman et al. 2011) and are more likely to use emergency services. Studies have also shown correlations between low HL and a higher body mass index (Sharif and Blank 2010) as well as increased drug abuse and high-risk behaviour (DeWalt and Hink 2009). Thus, the fact that HL is believed to be an important aspect of health promotion in childhood and adolescence (Okan et al. 2017), and that it has become an important goal in health education in schools should be no surprise (Nash et al. 2021; Ormshaw et al. 2013).

### Individual skills and abilities related to health literacy

Now that the internet has become the most important source of general information, and also one of the main sources for health information (Marstedt 2018; Rossmann et al. 2018; Wartella et al. 2016; Zschorlich et al. 2015), especially for young people (Ortner et al. 2019), the use of information and communication technology (ICT) is clearly of relevance in HL. Adolescents must now be able to work with technology, think critically about media and science issues, and navigate through a variety of information tools and sources to get the information they need to make health-related decisions (Norman and Skinner 2006a). Norman and Skinner (2006b) defined these skills as eHealth literacy (eHL), and constructed the eHealth Literacy Scale (eHEALS) instrument (Norman and Skinner 2006a) for its measurement. The scale focuses exclusively on those skills that are necessary for internet use and for searching for and assessing health-related information on the internet (Norman and Skinner 2006b).

Considering the increased importance of the internet for health-related information retrieval, investigating the influence of students’ online reading behaviour is highly relevant when investigating eHL and subjective HL. As previous research for college students has shown, high levels of HL are positively related to how intensively health-related websites are frequented (Rosenbaum et al. 2018). Similar results were found by Ceylan et al. (2022) for adolescents, i.e. frequency of internet use was associated with levels of HL. Adolescents who use the internet regularly have a higher level of HL than those who do not. Ceylan et al. (2022) also found that adolescents who search for health information on the internet were more likely to have an adequate level of HL.

In addition, scholars have identified self-efficacy as a crucial individual factor influencing HL. Self-efficacy is needed to seek and use health-related information (Subramaniam et al. 2015). Various studies of both adults (Osborn et al. 2010) and children (Fretian et al. 2020, 8–12 years) and adolescents (Ceylan et al. 2022, 15–18 years; Loer et al. 2020, 14–17 years) have found that higher levels of self-efficacy are significantly related to higher levels of HL.

### Sociodemographic factors related to health literacy

Besides individual skills and abilities, previous research has also identified sociodemographic and cultural aspects as influencing HL (Bröder et al. 2017). We distinguish these factors in terms of those operating on the individual level and those operating as part of the family background. On the individual level, both age and gender are believed to be linked to HL (Sukys et al. 2019). In a sample of 2369 Lithuanian students from the seventh to tenth grade, the self-perceived HL of boys was significantly lower in all grades than that of girls. Furthermore, students in the ninth and tenth grade achieved a significantly higher HL level than those in the seventh or eighth grade. Boys in the seventh grade showed the lowest level, and the highest level was found among the ninth-grade girls (Sukys et al. 2019). Similar results were reported for a Finnish sample. Here, the HL level was significantly lower for boys than for girls and significantly lower for younger students (seventh grade) than for older students (ninth grade) (Paakkari et al. 2018).

Regarding family background factors, studies have found a significant link between children’s and adolescents’ migrant background and low levels of HL (Loer et al. 2020; Quenzel et al. 2015; Röthlin et al. 2013). Furthermore, results from studies on adult HL showed that individuals with a family language (language spoken predominantly in the family) which differs to the environment language are particularly affected by health inequalities (Kutner et al. 2006) and have lower levels of HL (Ng and Omariba 2014; Rowlands et al. 2017). This also applies to children and adolescents. Tenth-grade students in Canada whose family language was not English (environment language) had lower levels of HL than students speaking English at home (Wu et al. 2010). Similarly, Bollweg et al. (2021) showed that fourth-grade students who spoke only German at home (environment language) had higher levels of HL and health knowledge than students with another family language.

Finally, the cultural capital of the family (including its socioeconomic background) (Chisolm et al. 2014; Chisolm et al. 2015; Levin-Zamir et al. 2011) and, in particular, the number of books at home (as an indicator of cultural capital) (Melton and Caldwell 2022) has also been linked to HL.

### Research questions

In the present study, we examine the perception of students regarding their own HL. Furthermore, we investigate the interrelations between individual and sociodemographic factors, and subjective HL and eHL, by addressing the following research questions:

1. How high is adolescents’ subjective HL and eHL?
2. Which individual and sociodemographic factors are related to adolescents’ subjective HL and eHL?

## Methods

The present study was part of a larger project that aimed to promote HL among secondary school students aged from 12 to 14 years. In this intervention study, students in the sixth and seventh grade of Austrian academic high schools and middle schools were trained to find, understand and evaluate health information on the internet using an adaptive digital training program. The data for the present study were collected during the pre-test phase of the project, and this article only reports on the instruments and variables relevant for this piece of research.

### Participants

The sample consists of 544 students aged from 11 to 16 years (*M* = 12.9 years, *SD* = 0.88). Forty-six percent (*n* = 249) of the students were female, and 54% (*n* = 295) male. The data were collected in 14 Austrian secondary schools, two from the academic track and 12 from the non-academic track. At the time of data collection, 247 (45%) students were attending the sixth grade, and 297 (55%) students the seventh grade. A total of 534 students provided sufficient information to determine their migrant background, and 44% (*n* = 236) had a migrant background (i.e. both parents born outside Austria). Overall, 62% (*n* = 339) of the students spoke German as their family language. Of those with a migrant background, 23% (*n* = 54) spoke German as their family language.

### Instruments

A version of Norman and Skinner’s (2006a) eHealth Literacy Scale (eHEALS), translated by the project team, was used to assess students’ eHL. The eHEALS is a tool for the self-reporting of the individual skills needed in finding, assessing and using electronic health information on the internet and consists of eight statements. Responses are rated on a scale ranging from 1 = strongly disagree to 5 = strongly agree. To determine the eHL of respondents, we took the answers to the eight statements and computed the mean score. The higher the value, the better the eHL. Our translated version of the eHEALS was found to have an acceptable Cronbach’s *α* = .79.

An adapted short version of the German-language European Health Literacy Survey (HLS-EU) questionnaire with 13 items was used to assess subjective HL. This scale was developed and validated by LOGO Jugendmanagement and queraum (querraum kultur- & sozialforschung 2017). They gave us permission to use the instrument for our study. The items employed deal with the perceived difficulty of performing selected health-related tasks or activities. Responses are recorded on a five-point scale ranging from 1 = very difficult to 5 = very easy. The mean score for all items was computed and used to assess the level of subjective HL. The higher the value, the higher the level of subjective HL. In our sample, the adapted short version of the HLS-EU exhibits a good level of internal consistency (Cronbach’s *α* = .85).

The self-reporting tool WIRKALLr (Allgemeine Selbstwirksamkeitserwartung) (Schwarzer and Jerusalem 1999) was used to assess students’ self-efficacy. The 10-item questionnaire measures how optimistic students are in coping with a particular situation, i.e. their confidence in mastering a difficult situation, whereby success is attributed to one's own competence. The items are assessed on a four-point scale with response options ranging from 1 = disagree to 4 = agree. The test score is obtained by deriving a mean score for all items. The higher the score, the more optimistic or confident students are. In our sample, the WIRKALLr exhibits a good level of internal consistency with a Cronbach’s *α* = .89.

Students’ online reading behaviour was assessed using a scale from PISA—Programme for International Student Assessment (bifie - Bundesinstitut für Bildungsforschung 2009). Of the original seven items, only those four with a clear focus on the frequency of different reading activities (not those dealing with text production and communication) were used. Respondents were asked to indicate how often they perform the following activities: (1) reading online news, (2) using an online dictionary or online encyclopaedia, (3) searching the internet for information on a specific topic, and (4) searching the internet for practical information. Responses were recorded on a five-point scale ranging from 1 = never or almost never to 5 = several times a day. In our sample, the online reading behaviour exhibited a rather low level of internal consistency, with Cronbach’s *α* = .68.

All other information (gender, age, number of books at home, migrant background and family language) were collected through a paper-and-pencil questionnaire.

### Procedure

The selection of the schools followed a two-step process. Our goal was to cover rural as well as urban schools and academic and non-academic track secondary schools, because in Austria, the circumstances of urban and rural and academic and non-academic track, respectively, schools differ. Urban schools usually have higher rates of students with a migration background, higher diversity in socioeconomic and cultural background and more students in the classroom than rural schools. Furthermore, while there is no significant performance difference between urban and rural academic track secondary schools, in the non-academic track, students in urban schools perform significantly worse on achievement measures than students in rural schools (BMBWF 2021).

In a first step, we therefore decided to draw our sample from three different clusters. We listed all secondary schools in the Austrian province of Styria (*N* = 190) in three different Excel tables clustered according to school type (academic and non-academic track) and region (rural and urban). Table 1 contains all 18 secondary schools from the non-academic track in the urban area of the Styrian capital Graz. Table 2 contains all 142 secondary schools from the non-academic track in rural areas of Styria, and Table 3 contains all 30 secondary schools from the academic track (including both urban and rural areas of Styria). In each table, the schools are arranged alphabetically and numbered accordingly. The second step, namely the drawing of the sample, was done with Python 3 and the module "random". Three schools were randomly drawn from Table 1, nine schools from Table 2 and two schools from Table 3. While this ratio of schools is not representative of Styria, and non-academic secondary schools in urban areas account for only around 9.5% of Styrian secondary schools, in our study we put a special focus on supporting potentially struggling students from highly diverse backgrounds and, therefore, decided to target a sample with a higher proportion of schools from non-academic urban secondary schools (around 21%).

**Table 1:** Means and standard deviation of students' test results in subjective HL, eHL, online reading behaviour and self-efficacy

	*M*	*SD*	*n*	Min	Max
Subjective HL	3.79	.63	544	1.00	5.00
eHL	3.46	.77	544	1.00	5.00
Online reading behaviour	2.67	.91	444	1.00	4.00
Self-efficacy	3.21	.78	458	1.00	5.00

**Table 2:** Pearson correlations of online reading behaviour, self-efficacy, age, eHL and subjective HL

	Online reading behaviour	Self-efficacy	Age	eHL
Self-efficacy	.07			
Age	.10*	.00		
eHL	.19***	.04	−.09*	
Subjective HL	.21***	.13**	.06	.35***

* *p* < .05; ** *p* < .01; *** *p* < .001.

**Table 3:** Point biserial correlations of gender, family language, migrant background, eHL and subjective HL

	Gender^1^	Family language	Migrant background	eHL
Family language^2^	−.06			
Migrant background^3^	−.03	.73***		
eHL	.06	.03	.02	
Subjective HL	−.01	−.10*	−.14**	.35***

* *p* < .05; ** *p* < .01; *** *p* < .001.

^1^0 = male, 1 = female

^2^0 = German, 1 = other than German

^3^0 = no migrant background, 1 = migrant background (both parents are not born in Austria)

The data were collected between January and April 2019. The survey took place in the computer rooms of the respective school and was accompanied by two trained people from the project team at the university and one teacher. Sociodemographic information and data on HL, online reading behaviour and self-efficacy were collected through an online questionnaire. For each participating student, we obtained parental consent. The data were stored and processed anonymously.

### Data analysis

SPSS 26 was used for the statistical analysis. Kolmogorov–Smirnov tests for eHEALS, HLS-EU, WIRKALLr, online reading behaviour and students age were conducted in order to determine the distribution of the data. The tests showed a significant result (*p* < .001) for all scales, thus indicating that the data were not normally distributed. Even though the normality assumption was not confirmed, due to the central limit theorem (Bortz and Schuster 2010) and the sample size of this study (*n* = 544), we nevertheless performed parametric tests to answer our research questions. To control, we also conducted non-parametric test (e.g. Spearman correlations) which confirmed the results. Furthermore, in preparation for calculating correlations and hierarchical regression analyses, we analysed the data for eHEALS, HLS-EU, online reading behaviour, WIRKALLr and age for linearity. Results showed that the relationship of all variables involved in the mediation analysis was approximately linear. This was assessed by visual inspection of the scatterplots after LOESS smoothing. Spearman, Pearson and point-biserial correlations, as well as hierarchical regression analyses, were also conducted. For all statistical analyses, the threshold for statistical significance was set at *p* < .05.

## Results

### Students’ eHealth literacy and subjective health literacy

Students’ eHL was found to be rather high on average (Table 1). Forty-three percent (*n* = 262) of the students “strongly agreed” with the statement that they know how to use information from the internet for their own support. Of the eight eHEALS items, this item received the strongest level of approval with *M* = 4.08 (*SD* = 1.06). The lowest value (*M* = 2.9, *SD* = 1.27) was achieved for the statement regarding knowledge of web pages on health-related questions. Here, only 12% (*n* = 71) of the students “strongly agreed”.

With respect to subjective HL, the results were also above the scale mean. The item “How easy or difficult is it for you to find out what to do in an emergency?” obtained the highest value (*M* = 4.09; *SD* = 1.00). A total of 257 (43%) students stated that they found it easy to figure out what to do in an emergency. In contrast, the item “How easy or difficult is it for you to evaluate if you can trust information about health risks in the media?” achieved the lowest value of *M* = 3.30 (*SD* = 1.14). Twenty-four percent (146) of the students stated that they found such evaluation very difficult or difficult.

Regarding the relationship between eHL and subjective HL, Pearson correlations (Table 2) showed that both abilities were significantly related to each other (*r*_(542)_ = .35, *p* < .001). This indicates that students with higher eHL also have a higher subjective HL, and vice versa.

### Bivariate analyses: factors influencing health literacy

Concerning students’ self-efficacy, results showed a mean of *M* = 3.21 (*SD* = .78), which is above the mean of the norm sample (*M* = 2.93, *SD* = .07) (Hinz et al. 2006). A weak correlation between subjective HL and self-efficacy was observed (*r*_(456)_ = .13, *p* = .005). This leads to the assumption that students with higher self-efficacy also show higher subjective HL.

Students' online reading behaviour also showed a significant positive correlation with subjective HL (*r*_(442)_ = .21, *p* < .001) and eHL (*r*_(442)_ = .19, *p* < .001). These findings indicate that students who spend more time reading and searching for online information have higher levels of both subjective HL and eHL.

Regarding the relationship between students’ age and subjective HL, no significant correlation was found (*r*_(527)_ = −.06, *p* = 157). In contrast to this, the results of a Pearson correlation showed there to be a significant negative relationship between eHL and students’ age (*r*_(527)_ = −.09, *p* = .041).

Spearman’s rank correlation was computed to assess the relationship between the number of books in the household and subjective HL and eHL. The results showed a significant correlation between subjective HL and the number of books at home (*r*_(542)_ = .15, *p* < .001). This indicates that students with more books at home have a higher level of subjective HL. However, no significant correlation was found between the eHL and the number of books at home (*r*_(542)_ = .01, *p* = .756).

Furthermore, as seen in Table 3, we conducted point-biserial correlation analysis to determine relationships between the subjective HL, eHL and the dichotomous variables (gender, family language, migrant background). This analysis showed no significant correlation between gender and subjective HL (*r*_(542)_ = −.01, *p* = .762), nor between gender and eHL (*r*_(542)_ = .06, *p* = .170).

Neither could we find a significant correlation between students’ family language and eHL (*r*_(542)_ = .03, *p* = .527). However, the results showed a negative significant relationship between family language and subjective HL, which indicates that students with German as their family language have higher levels of subjective HL (*r*_(542)_ = −.10, *p* = .017).

Finally, regarding the relationship between migrant background and eHL, no significant correlation was found (*r*_(542)_ = .02, *p* = .581). In contrast to this, however, a negative significant relationship was found between migrant background and subjective HL (*r*_(542)_ = −.14, *p* = .002). These findings imply that students with no migrant background have a higher level of subjective HL.

### Hierarchical regression analyses: factors predicting health literacy

To examine which variables predicted students’ subjective HL and eHL, we conducted hierarchical regression analysis. Before computing the models, key requirements for performing the regression analyses were tested. Homoscedasticity and normal distribution of the residuals were verified through visual examination. The absence of multicollinearity was tested through the variance inflation factor (VIF). The absence of autocorrelation was confirmed by means of a Durbin–Watson test, resulting in a value of 1.65.

Furthermore, in preparation for the calculation of the regression analysis, the ordinal variable for the number of books at home was dummy coded. This meant that two new variables were created.

The first regression analysis (Table 4) was conducted to examine which variables predicted students’ subjective HL. The predictor variables were added block-wise to the model. In the first model, we added the variables gender (0 = male, 1 = female) and age. These represent the students' individual characteristics.

**Table 4:** Summary of hierarchical regression analysis for variables predicting subjective HL (*N* = 361)

	Model 1				Model 2				Model 3			
	*B*	*SE B*	*β*	*t*	*B*	*SE B*	*β*	*t*	*B*	*SE B*	*β*	*t*
Age	−.109	.037	−.153**	−2.92	−.090	.039	−.126*	−2.30	−.113	.038	−.158**	−2.94
Gender	−.121	.064	−.099	−1.90	−.121	.065	−.099	−1.87	−.137	.063	−.112*	−2.17
Migrant background					−.075	.102	−.061	−.74	−.061	.099	−.050	−.62
Family language					−.068	.102	−.054	−.67	−.056	.099	−.044	−.56
Books at home I (26–100 books)					−.026	.079	−.020	−.33	−.064	.078	−.049	−.83
Books at home II (over 100 books)					−.018	.084	−.013	−.21	−.032	.082	−.023	−.39
Online reading behaviour									.159	.035	.235***	4.57
Self-efficacy									.027	.041	.034	.65
*R*^2^		.030				.040				.095		
Adjusted *R*^2^		.025				.024				.075		

*p < .05; **p < .01; ***p < .001.

Model 1 (*F*_(2,358)_ = 5.55; *p* = .004) explained 2.5% of the variance in subjective HL (adj. *R*^2^ = .025). As seen in Table 4, only students’ age was a significant predictor (β = −.153, *p* = .004). These results indicate that younger students have a higher level of subjective HL than older students. In Model 2, we added further potential predictors related to the sociodemographic characteristics of the students. Here, the variables migrant background (0 = no migrant background, 1 = migrant background; defined as both parents born outside Austria), students’ family language (0 = German, 1 = other) and the two dummy coded variables for number of books at home (variable 1: 0 = less than 26 books, 1 = between 26 and 100 books at home, 0 = more than 100 books; variable 2: 0 = less than 26 books, 0 = between 26 and 100 books, 1 = more than 100 books) were entered in a further step. While the results for Model 2 were also significant (*F*_(6,360)_ = 2.45; *p* = .025), the model led to no increase in explanatory power (adj. *R*^2^ = .024). As in Model 1, only age was found to be a significant predictor of students' subjective HL (β = −.126, *p* = .022).

Finally, the variables for students’ online reading behaviour and self-efficacy were additionally integrated into Model 3. This resulted in a significant increase in variance explanation, which rose to 7.5% (*F*_(8,360)_ = 4.63; *p* < .001). This is mainly due to the variable online reading behaviour, which was the only highly significant predictor (β = .235, *p* < .004) and therefore the most influential predictor in our model. According to this result, students who spend more time searching for and reading information online have a higher level of subjective HL. Furthermore, as in the previous models, age was also a significant predictor in Model 3 (β = −.158, *p* = .004). Additionally, gender became a significant predictor of subjective HL (β = −.112, *p* = .031), indicating that boys have a higher level of subjective HL than girls.

A second regression analysis (Table 5) was conducted in order to examine which variables predicted students’ eHL. Again, the predictor variables were added block-wise to the model. As in the first regression analysis, students' age and gender are included in Model 1. Model 1 (*F*_(2,360)_ = 5,29; *p* = .005) explained 2.3% of the variance in eHL (adj. R^2^ = .023), but only students’ age was a significant predictor (β = −.170, *p* = .001). Again, as in the first regression analysis, students’ socioeconomic characteristics (migrant background, family language and number of books at home) were added to Model 2 (*F*_(6,360)_ = 2,62; *p* = .017). The results for Model 2 were also significant, and there was a slight increase in explanatory power to 2.6% (adj. *R*^2^ = .026). As in Model 1, only age was a significant predictor of eHL (β = −.193, *p* < .001).

**Table 5:** Summary of hierarchical regression analysis for variables predicting eHL (*N* = 361)

	Model 1				Model 2				Model 3			
	*B*	*SE B*	β	*t*	*B*	*SE B*	β	*t*	*B*	*SE B*	β	*t*
Age	−.154	.047	−.170**	−3.25	−.174	.049	−.193***	−3.53	−.196	.049	−.217***	−3.99
Gender	−.023	.081	−.015	−.28	−.010	.082	−.006	−.12	−.022	.081	−.014	−.28
Migrant background					.056	.129	.036	.44	.064	.127	.041	.508
Family language					.074	.128	.046	.58	.084	.127	.052	.66
Books at home I (26–100 books)					.075	.100	.045	.75	.042	.099	.026	.43
Books at home II (over 100 books)					−.105	.106	−.060	−.99	−.177	.105	−.067	−1.12
Online reading behaviour									.162	.044	.190***	3.65
Self-efficacy									−.011	.052	−.011	−.21
*R*^2^		.029				.043				.077		
Adjusted *R*^2^		.023				.026				.057		

**p < .01; ***p < .001.

Finally, after inclusion of students’ online reading behaviour and self-efficacy, Model 3 (*F*_(8,360)_ = 3.70; *p* < .001) explained 5.7% of the variance in eHL (adj. *R*^2^ = .057). As before, age was a significance predictor (β = −.217, *p* < .001). Additionally, students’ online reading behaviour also became a highly significant predictor (β = −.190, *p* < .001). These results indicate that both age and online reading behaviour are influential predictors of eHL. Thus, younger students and those who spend more time searching for and reading information online rate their eHL higher. However, the online reading behaviour of the students contributes significantly to the explained variance and can therefore be seen as the most important predictor of eHL.

## Discussion

In this paper, we examined factors predicting subjective HL and eHL in a sample of 544 secondary school students aged from 11 to 16 years. The different individual and sociodemographic factors believed to be important for HL were analysed.

Regarding research question 1—students’ self-assessment of HL—our results revealed that students' subjective HL and eHL are rather high on average. These results are in line with findings from other international studies on subjective HL (Fretian et al. 2020) and eHL (Ghaddar et al. 2012; Koo et al. 2012) of children and adolescents. In particular, our findings are similar to those of a study on Serbian middle school students’ eHL (Gazibara et al. 2019). In the Serbian study, adolescents also reported that they lack knowledge concerning which web pages are available for health-related questions. High confidence was also observed with regard to knowing how to use internet information for help. This is also in line with our findings, as 43% (*n* = 262) of students strongly agreed with this statement.

Furthermore, we were also able to demonstrate that subjective HL correlates positively with eHL. Considering that HL is one of the six core skills of eHEALS (Norman and Skinner 2006a), it is not surprising that these two competencies correlate with each other in our sample. Similar results have been found elsewhere, particularly in studies of adults (Del Giudice et al. 2018; Duplaga et al. 2017). Self-assessment instruments for subjective HL were found to be related to eHL as measured by eHEALS. In our study, we were able to confirm this association for children and adolescents.

Bivariate and hierarchical regression analyses were performed in order to address our second research question: “Which individual and sociodemographic factors are related to adolescents’ subjective HL and eHL?” Regarding students’ self-efficacy and its potential influence on HL, our results are particularly interesting as they contradict results from previous research. Initially, a weak significant correlation between self-efficacy and subjective HL, but not for eHL, was found in the bivariate analysis. However, regression analysis found no significant association between self-efficacy and subjective HL, nor between self-efficacy and eHL. Previous studies conducted with a similar age group, and using some of the same instruments, showed that self-efficacy was a significant predictor of both subjective HL (Ceylan et al. 2022; Fretian et al. 2020; Loer et al. 2020) and eHL (Ghaddar et al. 2012). This discrepancy is interesting because instruments that measure HL through self-report questionnaires are often criticized for measuring self-efficacy or health-related behaviour rather than HL (Frisch et al. 2012). Should that be the case, then a significant correlation between self-efficacy and HL should also be detectable in our study. However, it is possible that the influence of self-efficacy is negligible in our sample because other psychological factors are more important. As Gerich and Moosbrugger (2018) stated, subjective instruments for assessing HL are influenced, amongst other things, by knowledge, empowerment and trust in the health system, as well as by self-efficacy. These factors need to be investigated in further research.

Bivariate analyses revealed weak effects concerning the relationship between migrant background, family language and subjective HL. The results showed that students with no migrant background, and students who speak German with their families, have higher levels of subjective HL. With respect to eHL, such a correlation was not found in the bivariate analyses. Furthermore, regression analysis showed that migrant background and family language are not significant predictors of subjective HL and eHL. These findings are partly in line with previous research (family language: Fretian et al. 2020; Quenzel et al. 2015; Röthlin et al. 2013; migrant background: Röthlin et al. 2013), although other studies did find a significant association between migrant background and HL (Loer et al. 2020) and between family language and HL (Bollweg et al. 2021; Wu et al. 2010). These discrepancies may be due to the fact that children and adolescents tend to provide socially desirable answers (Domanska et al. 2018), have little experience in navigating within the health system, generally have greater trust in the health system, are often unaware of potential difficulties, and thus overestimate their own competencies (Gerich and Moosbrugger 2018). Thus, their self-assessed HL may not accurately reflect their actual abilities (Taba et al. 2022).

Looking at HL over time, subjectively assessed HL has been found to decrease with age (Sørensen et al. 2015), which means that people become more realistic in their self-assessment as they get older. This could also explain, in contrast to other studies (Paakkari et al. 2018; Sukys et al. 2019), the negative correlation between HL and age that was found in our study. Several studies have shown that adolescents tend to overestimate their self-assessed HL more than adults (Firnges et al. 2016; Röthlin et al. 2013), and that the phenomenon of overestimating oneself is even more pronounced in younger children (Blatchford 1992; Shin et al. 2007). This may also be due to the fact that young people have little contact with the healthcare system or with managing diseases, as they are, for the most part, in good health, and thus tend to overestimate their competencies (Firnges et al. 2016). However, whether there is also a difference between different age groups among adolescents has not yet been investigated and should thus be considered in future studies.

Regarding the cultural capital of the family, we investigated the number of books at home as an indicator of cultural capital and a potential influencing factor. While Melton and Caldwell (2022) found that a higher number of books at home was associated with higher levels of HL, we were not able to find similar results. Commonly, rather than focusing specifically on the number of books at home, studies (e.g. Chisolm et al. 2014, 2015; Levin-Zamir et al. 2011) used students’ socioeconomic background in order to determine the influence of cultural capital on HL. Perhaps, in order to get a broader understanding of the actual impact on HL, it would be more appropriate in future studies to take the number of books into account.

While there is broad agreement on gender differences in HL among adults (Griebler et al. 2021; Lee et al. 2015; Sørensen et al. 2015), the results regarding children and adolescents have not been so clear. When gender differences are identified, they usually indicate that girls have higher levels of HL than boys (Chang et al. 2016; Fleary et al. 2018; Levin-Zamir et al. 2011; Paakkari et al. 2018; Page et al. 2011; Sukys et al. 2019). One exception was Chang (2011), which found significantly higher HL for boys. While these findings are generally in line with our results, we only found a gender difference in favour of boys with respect to subjective HL. However, this difference only became visible in the third regression model, and it has less explanatory power than the other two highly significant predictors, age and online reading behaviour. Nevertheless, as results for adults show, gender differences develop during adolescence. Future studies should thus investigate when and under what conditions such differences in HL emerge.

Even though the explanatory power (adj. *R*^2^) of the two calculated regression models is low, our study could reveal some interesting findings that could be relevant for future studies. One of the most interesting findings of our study, and one which is in contrast to those of other studies, is that sociodemographic factors are not associated with children’s and adolescents’ HL (Bröder et al. 2017). Instead, students’ online reading behaviour was identified as the most influential predictor of both subjective HL and eHL. As previous research has shown, high levels of HL are positively related to frequency of use of health-related websites (Rosenbaum et al. 2018), and to how often people engage in searching for health-related information on the internet (Ceylan et al. 2022). As confirmed by our results, how much time children and adolescents spend searching for and reading information on the internet has a significant impact on their HL.

Reasons why children and adolescents search for health-related information on the internet also have an impact on their HL. For example, Ghaddar et al. (2012) showed, that children and adolescents who searched for information about a family member's health had higher levels of eHL. Young people in particular use the internet to search health-related information when they are dealing with a sensitive topic that they find difficult to discuss with another person (Eysenbach 2007; Park and Kwon 2018). Therefore, the internet provides an anonymous environment to obtain information on various topics (Lupton 2021) and is one of the most important sources for health-related information (Marstedt 2018; Wartella et al. 2016; Zschorlich et al. 2015). This has become even more true since the beginning of the COVID-19 pandemic (Badell-Grau et al. 2020; Bento et al. 2020). Thus, programs for promoting HL among children and adolescents should be designed so as to foster those skills required for effective internet use. In addition, especially in the context of the increase in misinformation on the internet related to the COVID-19 pandemic (Zarocostas 2020), such programs should support the development of skills that children and adolescents need to not only find and understand health information from the internet, but also to critically evaluate and process it. Schools provide an ideal setting for such programs (Taba et al. 2022), as they can target measures for reaching all children and adolescents. It is important that children and young people are regularly tasked with gathering (health-related) information on the internet. When undertaken in a school environment, individuals gain valuable experience, are made aware of their capabilities and limitations, and can thus learn to better assess their own competencies.

### Limitations

Owing to the limitations of our study, a certain amount of caution is needed when interpreting the above results. As the instruments used to assess HL (eHEALS for eHL and HLS-EU for subjective HL) are based on self-assessment, augmenting such measures by the addition of instruments based on peer assessment is likely to be helpful, and may even lead to a more differentiated picture. The limitations of the sample used may also mean that results are not easily generalized. The sample was taken from a longitudinal intervention study. It is likely that this may have introduced an inherent element of bias owing to the process of self-selection among schools; i.e. those schools which already recognized the necessity of such intervention may have been relatively more willing to participate in the intervention program.

Furthermore, since the data was collected before the COVID-19 pandemic, the results might be different now. As shown in other studies, the search for health-related information on the internet increased during the pandemic (Badell-Grau et al. 2020; Bento et al. 2020; Dadaczynski et al. 2021; Vismara et al. 2021). As a result, people may have more experience of searching for health-related information on the internet, and may estimate their competencies higher.

Finally, even though we included various sociodemographic and individual factors in our regression models, the explanatory strength in our two models is very low. Therefore, it would be advisable for further studies to include other variables (e.g. parents’ education level, family affluence) that have been shown to be significant predictors in other studies (Dağhan et al. 2022; Fretian et al. 2020).

### Conclusions

This is one of the first studies to examine the relationship between individual and social factors and subjective HL and eHL among secondary school students in Austria. The results show that students’ online reading behaviour and age are significant for HL. Early promotion of HL is necessary in childhood and adolescence in order to facilitate health equity. This means that school-based HL programs need to foster skills which allow students to make effective and critical use of internet information. This entails an interdisciplinary approach, and such programs should not be confined to media studies alone. The same is true for health education. Health education is also an interdisciplinary area. In the current age of digitalization, critical media literacy and HL are intertwined, and teachers should take the opportunity to address related issues regularly and across disciplinary boundaries.

## Data Availability

Data and material are not available for third parties.

## Code availability (software application or custom code)

Statistical analyses were conducted using SPSS 26. The statistical codes used will be available from the corresponding author.
